# Myeloperoxidase: Regulation of Neutrophil Function and Target for Therapy

**DOI:** 10.3390/antiox11112302

**Published:** 2022-11-21

**Authors:** Salma A. Rizo-Téllez, Meriem Sekheri, János G. Filep

**Affiliations:** 1 Department of Pathology and Cell Biology, University of Montreal, Montreal, QC H3T 1J4, Canada; 2 Research Center, Maisonneuve-Rosemont Hospital, Montreal, QC H1T 2M4, Canada

**Keywords:** neutrophil, myeloperoxidase, degranulation, neutrophil extracellular traps, host defense, autoimmunity, inflammation

## Abstract

Neutrophils, the most abundant white blood cells in humans, are critical for host defense against invading pathogens. Equipped with an array of antimicrobial molecules, neutrophils can eradicate bacteria and clear debris. Among the microbicide proteins is the heme protein myeloperoxidase (MPO), stored in the azurophilic granules, and catalyzes the formation of the chlorinating oxidant HOCl and other oxidants (HOSCN and HOBr). MPO is generally associated with killing trapped bacteria and inflicting collateral tissue damage to the host. However, the characterization of non-enzymatic functions of MPO suggests additional roles for this protein. Indeed, evolving evidence indicates that MPO can directly modulate the function and fate of neutrophils, thereby shaping immunity. These actions include MPO orchestration of neutrophil trafficking, activation, phagocytosis, lifespan, formation of extracellular traps, and MPO-triggered autoimmunity. This review scrutinizes the multifaceted roles of MPO in immunity, focusing on neutrophil-mediated host defense, tissue damage, repair, and autoimmunity. We also discuss novel therapeutic approaches to target MPO activity, expression, or MPO signaling for the treatment of inflammatory and autoimmune diseases.

## 1. Introduction

Neutrophil granulocytes are the most abundant immune cells, constituting about 60–70% of all leukocytes in human blood. Neutrophils are typically the first immune cells to be recruited to sites of infection and tissue injury [1,2,3], and provide the first line of defense to the host against invading pathogens [2,4,5]. This role is best exemplified by the susceptibility of neutropenic patients to repeated bacterial infections [6]. Neutrophils can efficiently kill bacteria through different defense mechanisms [2,7,8], including the mobilization and discharge of microbicide proteins and proteases stored in pre-formed cytosolic granules [9,10]. Granules can fuse with pathogen-containing phagosomes following phagocytosis of bacteria or the plasma membrane in response to pathogens that cannot be phagocytosed, leading to the extracellular release of their content [2,8]. Among the microbicide proteins is the heme-containing enzyme myeloperoxidase (MPO), the most abundantly expressed protein by human neutrophils. MPO utilizes chloride as a co-substrate with H_2_O_2_ to generate chlorinating oxidants, such as HOCl, which have one of the highest cytotoxic potentials to bacteria and living cells [11]. However, the release of some neutrophil products, including MPO, to the extracellular milieu can be detrimental to the host by inflicting tissue damage and consequently exacerbating inflammation [5,11,12]. Neutrophil-driven inflammation has been recognized as a common mechanism underlying a wide range of pathologies, including atherosclerosis, cardiovascular, respiratory, autoimmune and neurodegenerative diseases, sepsis, and cancer [5,13]. Elevated plasma MPO levels are frequently detected in patients under these conditions and correlate with disease severity [14,15,16,17,18]. By contrast, recent studies in MPO-deficient animals reported exaggeration of the inflammatory response reviewed in [19], implying protective functions for MPO. Given these diverse effects, the balance between MPO’s deleterious and beneficial effects will likely determine its contribution to the outcome of the inflammatory response. 

The prevailing and rather simplistic view of MPO as a cytotoxic agent has undergone substantial revision in the past decade, and novel paradigms have emerged. It is now apparent that the roles of MPO extend beyond its antimicrobial function and inflict bystander tissue damage to the host. Accumulating evidence indicates that MPO can directly modulate function of inflammatory and other cells, including neutrophils, macrophages and dendritic cells, endothelial and epithelial cells, and shape immunity [20,21]. Of particular importance, MPO modulates the functions of neutrophils, which interact with other innate and adaptive immune cells to orchestrate immune responses [13]. In this paper, we outline recent advances in the multifaceted roles of MPO in immunity, focusing on its participation in the regulation of neutrophil function, repair, and return to homeostasis to complement other in-depth reviews [11,19,22,23,24]. We will also discuss the therapeutic potential of targeting MPO or MPO signaling for the treatment of inflammatory diseases. 

## 2. Myeloperoxidase Expression and Release

MPO, a member of the heme peroxidase-cyclooxygenase superfamily [25], is abundantly expressed in neutrophil granulocytes, and to a lesser extent in monocytes and a subset of macrophages [11,26,27]. Under pathological conditions, neurons, astrocytes, and endothelial cells were also found to express MPO [28,29,30,31,32]. MPO is encoded by a single gene located on chromosome 17, segment q12-24. MPO transcription is driven by C/EBPα, Runx1, and GATA-family transcription factors [33]. The primary ~80 kDa translation product is an inactive enzyme that undergoes co-translational N-glycosylation and heme incorporation to generate enzymatically active proMPO, followed by enzymatic removal of the propeptide before final proteolytic processing in primary or azurophilic granules [22]. These granules are the first to be formed during myelopoiesis (hence their name) and stain by the dye azure A (i.e., azurophilic). In addition to MPO, they also contain multiple antimicrobial proteins, including serine proteases such as elastase, proteinase 3, cathepsin G, and azurocidin, membrane permeabilizing proteins such as lysozyme and Cap57 (bactericidal/permeability-increasing protein, BPI) and α-defensins [9,34]. Azurophil granules are heterogenous in their protein content and sub-cellular targeting [35,36,37]. For instance, the generation of defensin-rich versus defensin-poor or secretory versus non-secretory azurophilic granules was described [37], though the functional importance of these differences remains to be established. 

The release of granule contents (degranulation) occurs in hierarchical order in a step-wise process and depends on specific signaling events. Ligation of cell surface chemotactic or phagocytic receptors [38] or the intracellular receptor TLR9 [39] triggers activation of calcium-dependent and Hck-dependent signaling pathways, Rac-2-mediated actin and microtubule reorganization that transport azurophilic granules to the plasma membrane or the phagosome and dock them to the target membrane. Tethering and then fusion of azurophilic granules with the target membranes is driven by Rab27a and its effector Slp1/JFC1, followed by the calcium-dependent formation of a SNARE complex consisting of the granule v-SNARE protein VAMP-7 and its cognate SNAP-23/syntaxin4 complex on the cell membrane [38,40]. Azurophilic granules that do not express Rab27a and Slp1/JFC1 mobilize specifically to phagosomes [41]. Finally, expansion of the fusion pore assures releasing of granule contents.

The mature MPO is a homodimer formed by two glycosylated protomers that contain a light chain of 14.5 kDa, a heavy chain of 58.5 kDa, and a heme group [42,43,44]. Each subunit, known as hemi-MPO, is functionally independent [45,46]. However, some proMPO may escape granule targeting and will be secreted to the extracellular space as a monomer [22]. Furthermore, transcription of the MPO gene in foam cells, which lack the machinery to proteolytically process proMPO, results in the release of proMPO through the secretory pathway [22,23]. 

MPO together with lactoferrin, lysozyme, cathepsin G, and α-defensin was also detected in thin cellular protrusions (termed cytonemes), which have been proposed to deliver signaling proteins to neighboring cells [47,48] as well as in extracellular vesicles, another vehicle of intercellular communications [49,50]. The mechanisms by which MPO is targeted to these structures remain to be investigated.

Hereditary MPO deficiency appears approximately 1 in 2000–4000 in Europe and North America, less common in the Japanese population [51]. Severe MPO deficiency is associated with a higher incidence of bacterial and fungal infections [11]; however, the majority of MPO deficiency cases are thought to be subclinical [52]. Subjects carrying the G-463A MPO polymorphism have higher levels of total and low-density lipoprotein-associated cholesterol, and elevated risk for coronary artery disease [53], MPO-ANCA vasculitis [54], lung [55,56], and pancreas cancer [57].

## 3. Enzymatic Properties

The structure and enzymatic properties of MPO have been well characterized and discussed in previous in-depth reviews [11,23]. MPO contains distinct prosthetic groups. The secreted form of the enzyme, MPO-Fe (III) reacts in a rapid and reversible manner with H_2_O_2_ to form Compound I, a two-electron oxidized intermediate with a Fe(IV)=O group. This redox intermediate oxidizes halides (Cl^−^, Br^−^) and pseudohalides, e.g., SCN^−^) in the presence of H_2_O_2_ to their corresponding hypohalous acids [11]. This pathway is referred to as the halogenation cycle with Cl^−^ as the preferred substrate and hypochlorous acid (HOCl) as the dominant oxidant produced under physiological conditions. These reactive oxygen intermediates are generally considered components of the intracellular and extracellular microbicidal defense [11,24]. Of note, high concentrations of H_2_O_2_ can inactivate MPO. 

MPO also catalyzes typical peroxidation reactions [11]. In the peroxidation cycle, Compound I can oxidize a number of physiological substrates through two sequential one-electron steps, forming Compound II and MPO-Fe (III), respectively. The conversion of Compound II to MPO-Fe (III) is the rate-limiting step of the peroxidase cycle. An additional compound, Compound III (or MPO-Fe (II)-O_2_) may be produced with an extra one-electron reduction or with interaction with O_2_^•−^. At high H_2_O_2_ concentrations, the formation of Compound III is an integral part of the catalytic cycle. A second reaction with O_2_^•−^ will turn Compound III in to the enzyme native state. 

HOCl readily reacts with sulfur and nitrogen atoms, in particular glutathione and proteins containing methionine or cysteine. HOCL oxidation of Met residues can lead to the inactivation of protease inhibitors, proteinases, growth factors, and lysozyme, and modulation of NF-κB activation through the oxidation of I-κB. Moreover, the reaction of HOCl with Cys residues could inactivate enzymes, such as creatine kinase and glyceraldehyde-3-phosphate dehydrogenase (GAPDH), while enhancing the activity of matrix metalloproteinases [11,23]. In addition, the HOCl-mediated oxidation of GSH into glutathione sulfonamide (GSA) would lead to impaired cellular redox homeostasis [58]. At low Cl^−^ concentrations, MPO can use O_2_^•−^ as a major co-substrate, switching to superoxidase activity [11]. 

## 4. MPO Regulation of Neutrophil Trafficking

In addition to the intracellular killing of invading pathogens, accumulation evidence indicates an important role for MPO in the regulation of neutrophil trafficking. Neutrophil recruitment is a multistep event allowing capture, adhesion, and extravasation of the cell [1,59]. Non-activated neutrophils adhere to MPO-coated surfaces [60], whereas “free” circulating MPO binds to the β_2_ integrin CD11b (Mac-1) [61] and modulates intracellular Ca^2+^ levels and cytoskeleton organization [62]. MPO was found to evoke highly directed neutrophil motility in vitro and to attract neutrophils in preclinical models of hepatic ischemia-reperfusion and cremaster muscle inflammation through its cationic surface charge independent of its catalytic properties [63]. Furthermore, catalytically inactive MPO reduced the thickness of the endothelial glycocalyx through ionic interaction with heparan sulfate side chains and induced shedding of the endothelial glycocalyx core protein syndecan-1 [64]. Administration of human MPO was associated with increased neutrophil accumulation in carrageenan-induced acute lung injury in mice [65]. These effects of MPO are consistent with providing adhesive support for neutrophils. Increased MPO expression was detected on the surface of neutrophils from patients with various inflammatory diseases, including sepsis, ischemia-reperfusion injury, or acute coronary syndromes, and correlated with plasma MPO levels [61]. By contrast, reduced neutrophil MPO expression has been suggested as being a good predictor of a higher risk of mortality in patients with sepsis [66].

Studies on MPO-deficient mice yielded apparently contradictory results. In some studies, genetic deletion of MPO was found to attenuate neutrophil accumulation and distant organ damage after renal ischemia-reperfusion [67], to reduce *E. coli* septicemia-induced pulmonary bacterial colonization, lung injury, and mortality [68], and to increase cellular protection in ischemic stroke [69]. Furthermore, MPO deficiency was associated with increased glomerular accumulation of neutrophils and induction of CD4+ T cell autoimmunity in a model of lupus nephritis [70]. Of note, MPO deficiency is associated with upregulated baseline expression of inducible NO synthase and increased NO production in the lung, which may partially compensate for the lack of HOCl-mediated bacterial killing [68]. The contribution of enhanced NO production to the protection afforded by MPO deficiency remains to be investigated. By contrast, other studies reported a role for MPO to limit excessive neutrophil accumulation. Thus, MPO-deficient mouse neutrophils display increased surface expression of CD11b and a pro-migratory phenotype in ischemia-reperfusion-induced liver injury [71]. Blockade or genetic deletion of MPO was found to significantly increase endotoxemia-associated mortality, whereas adoptive transfer of wild-type neutrophils protected against mortality [72]. Whether these actions were mediated by CD11b or ionic interactions remains to be investigated. MPO was shown to limit local tissue damage by converting diffusible H_2_O_2_ into highly reactive, but locally confined HOCl [73]. MPO may also modulate the inflammatory response through catalyzing the oxidation of lipid mediators [74], as exemplified by elevated plasma levels of cysteinyl leukotrienes and reduced oxidative metabolites of linoleic acid [75]. Another study has suggested that enhanced adhesive interactions were responsible for increased neutrophil migration into the peritoneal cavity in response to zymosan and the inflamed cremaster muscle in MPO-KO mice [76]. Consistently, the administration of recombinant MPO to wild-type mice diminished neutrophil migration and accumulation in the same models [76]. Genetic MPO deletion may have dual consequences; for instance, MPO deficiency prevented neutrophil-mediated renal injury, but aggravated T-cell immunity inducing crescentic glomerulonephritis [77].

Differences in the composition of primary neutrophil granules, in particular lack of defensins and low MPO content (about 10–20% of that of human neutrophils) in mouse neutrophils [78] should be considered for translating the data from mouse studies to the clinical setting. Furthermore, cell contact-dependent CD11b-mediated MPO transfer from neutrophils to endothelial cells can disrupt normal endothelial function [79], leading to endothelial damage and consequently enhanced leukocyte adherence. MPO delivered in extracellular vesicles might also evoke endothelial injury similar to that reported for epithelial cells [50].

## 5. MPO, Neutrophil Activation and Phagocytosis

MPO can regulate neutrophil function independent of enzymatic properties. MPO binding to CD11b on human neutrophils has been shown to evoke release of primary/azurophilic granule contents, including MPO and elastase, and to upregulation of CD11b expression through yet unidentified molecular mechanisms [61,65]. MPO ligation of CD11b leads to phosphorylation of p38 MAPK, ERK ½, and PI3K [61,65], and activation of NF-κB [61]. p38 MAPK induces phosphorylation of p47^phox^, the cytoplasmic regulatory subunit of NADPH oxidase [80], leading to superoxide formation [61], and transcription of NF-κB-regulated genes involved in the acute inflammatory response [81]. These findings are consistent with the function of CD11b as a bidirectional allosteric “signaling machine” [82], and CD11b-mediated outside-in signaling in degranulation [83]. These data also imply an MPO-centered feed-forward autocrine/paracrine mechanism for aggravation and prolongation of the inflammatory response, as exemplified in a mouse model of acute lung injury [65,84]. CD11b also functions as a receptor for complement C3b (CR3) that mediates phagocytosis of complement C3b-opsonized microbes and damaged cells [85]. MPO-deficient neutrophils exhibit higher levels of surface expression of CD11b and engulf more zymosan than do wild-type neutrophils [86]. Neutrophil-produced reactive oxygen and nitrogen species, such as H_2_O_2_ and ONOO^−^,have long been recognized as intracellular signaling molecules to regulate the activation of transcription factors, including NF-κB, and cytokine production [87,88]. Thus, it is plausible that MPO or MPO-derived oxidants HOCl (or its derivatives) function as signaling molecules to modulate CD11b-mediated phagocytosis. MPO deficiency may result in the accumulation of intracellular H_2_O_2_ [89], which together with reduced HOCl generation might lead to neutrophil activation via the NF-κB and ERK 1/2 pathways. Similar to H_2_O_2_, MPO-generated HOSCN targets cysteine residues present in protein tyrosine phosphatases, resulting in increased phosphorylation of p38 MAPK and ERK1/2, impaired antioxidant defenses and induction of pro-inflammatory cytokine gene expression and cytokine release [90,91]. MPO modulation of CD11b expression and/or function may vary on the stimulus and context. Indeed, studies comparing cytokine production of MPO-deficient and wild-type neutrophils revealed substantial stimulus-dependent variations. For instance, MPO-deficient neutrophils were found to produce less KC and MIP-1α, and higher amounts of IL-6, IL-10, and TNF-α than wild-type neutrophils challenged with LPS in vitro [92]. Stimulation of MPO-KO neutrophils with zymosan generated more IL-1α, IL-1β, MIP-1α, MIP-1β, MIP-2, and TNF-α as compared to wild-type neutrophils [93]. 

## 6. MPO Regulation of Neutrophil Lifespan

During migration from the blood to the inflamed tissue and within the inflammatory locus, neutrophils receive pro-survival cues that extend their lifespan by delaying intrinsic apoptosis [21,94]. CD11b-mediated binding of neutrophils to the endothelial counter-ligand ICAM-1 or fibrinogen induces activation of the PI3k/Akt, MAPK/ERK, and NF-κB signaling pathways [95,96,97], prevention of proteasomal degradation of the anti-apoptotic protein Mcl-1, a key regulator of neutrophil survival [98], and consequently suppression of caspase-3 activity. Adhesion per seis not a prerequisite for prolonged neutrophil survival [99] as ligation of CD11b with MPO or other soluble ligands also activates these signaling pathways [61,65] and generates survival signals for human neutrophils in vitro [65]. The survival signal does not require catalytic activity, for MPO inactivated with the suicide substrate 4-aminobenzoic acid hydrazide (4-ABAH) or the mechanism-based inhibitor 3-amino-1,2,4-triazol plus H_2_O_2_ exerted actions similar to that of native MPO [61,65]. Consistently, the administration of human MPO, resulting in levels similar to those detected in patients with inflammatory vascular diseases [14,15], prolongs the lifespan of circulating neutrophils through suppressing apoptosis in rats [65]. Furthermore, MPO suppresses neutrophil apoptosis and delays the spontaneous resolution of inflammation, resulting in the perpetuation of carrageenan-induced acute lung injury in mice [65]. These MPO actions closely resemble those of the pan-caspase inhibitor zVAD-fmk, which aggravates and prolongs carrageenan-elicited acute pleurisy [100] and lung injury [65]. Dissociation of the dimeric MPO into monomeric or hemi-MPO following reductive alkylation attenuates Ca^2+^ mobilization and degranulation, and results in a partial loss in prolonging neutrophil lifespan and attenuates degranulation [62]. These would imply a regulatory mechanism by which MPO may control the activation and fate of neutrophils. Elevated hemi-MPO levels were detected in the serum of patients with acute inflammation [62], suggesting that this regulatory mechanism may be operational in vivo. However, whether increased hemi-MPO levels were due to increased secretion or decomposition of MPO as well as the mechanism of MPO decomposition in the blood requires further studies. 

In contrast to generating survival cues in human neutrophils, MPO was reported to promote apoptosis in HL60 leukemia cells, and this was partially inhibited by the inactivation of MPO with 4-ABAH or methimazole [101,102]. While these studies did not address the involvement of CD11b in the pro-apoptotic action of MPO, it is plausible that ligation of CD11b exerts opposing actions in primary and leukemia cells likely by shifting the balance of pro-survival and pro-apoptosis cues. Interestingly, the pro-apoptotic action partially depends on the catalytic activity of MPO [101,102], whereas pro-survival signaling does not require catalytically active MPO [65]. Nevertheless, it is intriguing that MPO can prolong the lifespan of human neutrophils, the predominant source of this enzyme in spite of its potent cytotoxic properties [65].

## 7. MPO, NET Formation and Autoimmunity

Neutrophils can release extracellular traps (NET) to trap and kill pathogens extracellularly when phagocytosis is not feasible [8,103,104]. NETs are a meshwork of chromatin decorated with granule proteins, such as MPO, neutrophil elastase, and proteinase-3, and absorb pentraxin 3 [105] and complement [106]. In the suicidal pathway of NET formation (commonly referred to as NETosis), reactive oxygen species produced by NADPH oxidase via activation of the Raf-MEK-ERK and p38 MAPK pathways initiate NET extrusion. Neutrophil elastase translocates to the nucleus, where it partially degrades specific histones and synergizes with MPO in driving chromatin decondensation [107]. The action of MPO is independent of its enzymatic activity. Protein-arginine deiminase 4 (PAD4)-mediated chromatin decondensation ultimately leads to the extrusion of a DNA scaffold studded with citrullinated histones and cytotoxic granular proteins, including MPO [108,109]. Neutrophils from patients with complete MPO deficiency are unable to form NETs, which is associated with several microbial infections, especially with *Candida albicans* in a subgroup of these patients [51]. Partial MPO deficiency or pharmacological inhibition of MPO only delays and reduces NET formation [51]. The addition of exogenous MPO to MPO-deficient neutrophils does not rescue NET formation, suggesting that this process requires MPO translocation to the appropriate subcellular compartment. Vital NET release is independent of NADPH oxidase and occurs more rapidly than suicidal NET extrusion in response to *S. aureus*, *C. albicans*, *A. fumigatus*, Leishmania promastigotes [110,111], or anaplastic thyroid cancer cells [112] without compromising neutrophil viability. Some studies suggested a role for mitochondrial oxidants and selective extrusion of mitochondrial DNA in forming extracellular traps [113,114]. Thus, the molecular mechanisms governing the release of nuclear or mitochondrial DNA appear to differ [115]. The pathological relevance of suicidal versus vital NET extrusion to innate immunity and the role of MPO in this latter process require further studies. Previous studies showed that phagocytosis of bulky phosphatidylserine-decorated particles, such as apoptotic cells or activated platelets, rendered neutrophils unable to form NET [116,117]. Hence, modulation of phagocytosis by MPO (and other granule constituents) may represent another mechanism to regulate NET extrusion.

Apart from immune defense and infectious diseases, increasing evidence indicates that aberrant NET formation is a central event under a number of pathological conditions, including atherosclerosis, acute respiratory distress syndrome, rheumatoid arthritis, lupus erythematosus, metabolic syndrome, neurological disorders, and cancer [118,119]. NET biology has been the subject of several recent comprehensive reviews [118,119,120,121]. It is important to emphasize that NETs can exert both pro- and anti-inflammatory actions, even under the same pathological conditions, as shown, for example, in a model of rheumatoid arthritis [122]. NETs can limit inflammation by trapping and degrading cytokines and chemokines [123] and orchestrate induction and resolution of sterile crystal-mediated inflammation [124]. The role of MPO in these events is largely unknown. 

MPO has long been recognized as a target antigen in different forms of anti-neutrophil cytoplasmic antibody (ANCA)-associated vasculitides, microscopic polyangiitis, eosinophilic granulomatosis with polyangiitis and to a lesser frequency in granulomatosis with polyangiitis [125,126,127]. Externalization of MPO, together with other well-known antigens, such as double-stranded DNA and histones, through aberrant NET formation or impaired NET degradation, has been implicated in triggering autoimmunity in susceptible individuals [126,128]. Pathogenic ANCA bind to MPO expressed on the surface of cytokine-primed neutrophils, leading to their excessive activation [129]. The resulting necrotizing inflammation of small and medium blood vessels may affect various organs, including the airways, kidneys, skin, and the nervous system [127]. Furthermore, sera from patients with MPO-ANCA-associated microscopic polyangiitis [130] or systemic lupus erythematosus [128] show impaired capacity for NET degradation, partly due to lower serum DNase1 levels [130]. Sera from patients with MPO-ANCA-associated microscopic polyangiitis were found to have a high ability to induce NET formation [130]. These would likely form a vicious cycle that may contribute to disease progression. MPO has also been suggested to trigger autoimmunity during uncontrolled inflammation in mice [131], though it is unclear whether this process involves MPO binding to CD11b and/or NET formation. 

**Figure 1:**
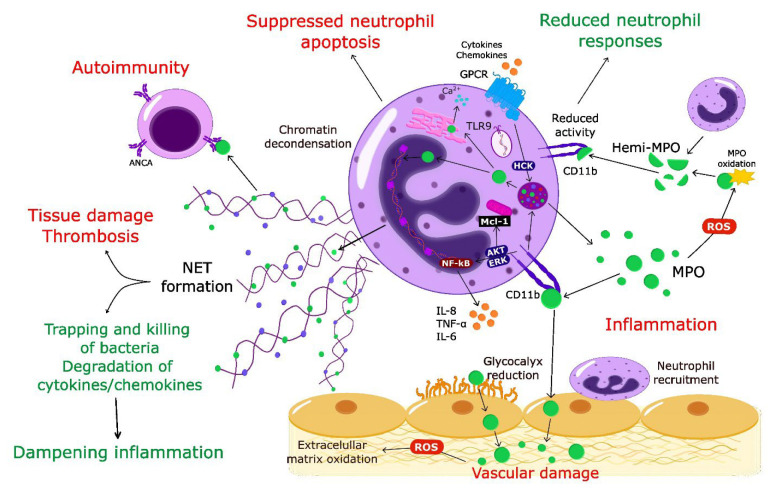
Multifaceted pro-inflammatory and protective actions of MPO. Activation of neutrophils through chemokine/cytokine receptors or TLR9 leads to release of MPO from primary granules MPO catalyzes local formation of highly toxic oxidants that contribute to tissue damage. MPO facilitates neutrophil trafficking into the inflamed site, evokes neutrophil activation, degranulation, and generates survival cues by suppressing constitutive neutrophil apoptosis through non-enzymatic actions on CD11b. MPO-CD11b form a feed-forward mechanism for perpetuating neutrophil-mediated inflammation. Dissociation of MPO into hemi-MPO results in reduced activity and blunted neutrophil responses. Intracellularly released MPO synergizes with neutrophil elastase to drive chromatin decondensation, leading to extrusion of neutrophil extracellular traps. NET-bound MPO contributes to bacterial killing and degrades cytokines/chemokines, thereby dampening inflammation. By contrast, NETs have been implicated in tissue damage and initiation of thrombosis. Aberrant NET formation (or impaired NET degradation) leads to prolonged presentation of MPO antigens, triggering autoimmunity. Red color indicates pro-inflammatory activities of MPO; green color refers to protective functions. ANCA, anti-neutrophil cytoplasmic antibody; GPCR, G protein-coupled receptor; NET, neutrophil extracellular trap; ROS, reactive oxygen species; TLR9, toll-like receptor 9.

Together with the changing perception of the role of neutrophils in homeostasis and pathogenesis [1,2,20,21], it has become apparent that MPO should no longer be considered exclusively as an enzyme-producing cytotoxic oxidant. Thus, in addition to inflicting tissue damage through enzymatic and non-enzymatic actions, MPO may also exert protective actions and contribute to the dampening of inflammation (Figure 1).

## 8. Myeloperoxidase as a Therapeutic Target

Considering the involvement of MPO in mediating neutrophil actions critical for host defense and pathological processes makes this protein an attractive therapeutic target. Indeed, while several potent MPO inhibitors have been developed in the last decade; limited information is available on the disease-modifying potential of blocking MPO’s enzymatic activities in preclinical models and patients [23]. Targeting the non-enzymatic properties of MPO or blocking MPO signaling has recently emerged as an alternative avenue to counter the deleterious actions of MPO. Table 1 summarizes selected approaches to inhibit MPO and to ameliorate its adverse effects.

## 9. Suppressing MPO Gene and Protein Expression 

While severe MPO deficiency is associated with an increased risk for infection [11], the incidence of cardiovascular diseases appears to be lower in MPO-deficient individuals as compared to the reference population [163]. Intriguingly, statins, which block 3-hydroxy-3-methylglutaryl CoA-reductase, were found to suppress MPO expression in macrophages at the gene and protein levels [164] and reduce serum MPO levels in diabetic hemodialysis patients [149] and patients with acute coronary syndrome [148]. Statin reduction of MPO may be mediated by blocking the production of isoprenoid intermediates of the mevalonate pathway, which is required for MPO expression [23]. Hence, it is plausible that some of the beneficial actions of statins can be attributed to reduced MPO levels or activity. Further studies should address the effects of statin in individuals with G-463A MPO polymorphism, a known risk factor for coronary artery disease [53]. 

All trans-retinoic acid (tretinoin), commonly used for the treatment of acute promyelocytic leukemia, is currently in a clinical phase 2 trial on the expression of the MPO (and protease 3) gene in patients with ANCA vasculitis. The patients will be followed for 12 months to assess changes in disease activity and incidence of disease relapse over a 12-month period [150]. No results have been posted to date.

## 10. MPO Inhibitors

The complexity of the catalytic mechanisms implies two major approaches for MPO inhibition. Irreversible inhibitors form strong covalent bonds with the iron atom in the heme center, thus blocking the reaction with H_2_O_2_ and rendering MPO inactive. Reversible inhibitors can form a complex with MPO, leading to blockade of the peroxidase cycle or act as a substrate for MPO, resulting in the accumulation of Compound II [11,23,165] Over the past three decades, several hydroxamic acids, benzoic acid hydrazides, indoles, and tryptamines have been identified as reducing substrates for Compounds I and II of heme peroxidases. 2(3*H*)-benzoxazolon derivatives, the antithyroid drugs methimazole and propylthiouracil, and dimethylthiourea were also reported to inhibit the peroxidase and chlorination activity of MPO. The characteristics and mechanisms of action of these compounds have been detailed in previous reviews [11,23,165]. For example, the suicide substrate 4-ABAH inhibits both peroxidation and chlorination, and its actions on neutrophils in vitro have been extensively characterized. The 2-thioxanthine class of irreversible MPO inhibitors shows the highest activity reported to date in inhibiting various MPO-catalyzed oxidative reactions in vitro [137,166]. 2-thioxanthines have modest effects on lactoperoxidase and thyroid peroxidase (no information is available on eosinophil peroxidase), raising the possibility of fine-tuning the specificity of mechanism-based inhibitors toward MPO. Among the irreversible inhibitors is melatonin, which in addition toits antioxidant properties also inhibits H_2_O_2_ consumption by MPO [165].

A growing body of preclinical and ex vivo experimental data suggests the beneficial actions of MPO inhibitors in attenuating inflammation and reducing tissue injury. For instance, MPO inhibitors were found to slow down the progression of atherosclerosis through preventing endothelial dysfunction and LDL oxidation in mice [132,134]. Pharmacological blockade of MPO activity also attenuated tissue injury and promoted resolution in murine models of pleurisy [167], immune complex vasculitis [144], and chronic obstructive pulmonary disease [138]. Moreover, MPO inhibition was reported to reduce neuroinflammation in specimens from patients with Parkinson’s disease [140] and to improve neurogenesis following ischemic stroke in mice [69].

There are several ongoing clinical trials to assess the safety and effectiveness of MPO inhibitors. Phase 1 and 2 trials with the orally active MPO inhibitor A (AZD4831) (Astra Zeneca) investigated its effectiveness in patients with heart failure with preserved left ventricular ejection fraction (HFpEF) [146]. Conventional therapies have limited success in this form of heart failure, which is frequently associated with neutrophilia and inflammation. Initial data indicated a good tolerability profile, though no information on the effects on heart failure has been posted to date. Promising preliminary results suggesting a reduction in neuro-inflammation, assessed by biomarkers of microglial activation, were reported from a randomized phase 2 trial with the MPO inhibitor AZD3241 (Astra Zeneca) in patients with multiple system atrophy [168]. A phase 3 trial has been accepted by the FDA. Results from a phase 1 trial indicated a good bioavailability and safety profile for another MPO inhibitor, PF-06282999 [169]. No information is available on further testing of this compound.

A double-blind randomized controlled trial investigated the effects of doxycycline on systemic inflammatory markers (cytokines and C-reactive protein) and neutrophil-specific markers, including MPO in the sputum of patients with stable chronic obstructive pulmonary disease (COPD) [170]. A three-week course of doxycycline failed to affect MPO in the sputum, serum inflammatory markers, and lung function parameters.

## 11. Inhibition of Granule Trafficking, Docking and Degranulation

Structural modeling and high-throughput screening analysis led to identifying a series of small molecules termed Nexinhibs (neutrophil granule-specific exocytosis inhibitors) [171]. Nexinhib20 was found to selectively interrupt the interaction of Rab27a with the effector Slp1/JFC1, which selectively regulates the release of the azurophilic granule cargo [38] without affecting phagocytosis, NET formation, or cell viability [151,171]. Treatment of mice with Nexinhib20 resulted in marked decreases in plasma levels of azurophilic granule proteins [172] parallel with attenuation of neutrophil accumulation in the kidney and liver during LPS-induced systemic inflammation [151]. These changes resemble those observed in LPS-challenged Rab27a-deficient mice [173]. Peptide aptamers derived from SNARE domains that compete for binding between intact SNARE proteins have also been developed [171]. The aptamers were fused with the cell-penetrating peptide HIV TAT to facilitate uptake by neutrophils [171]. However, the aptamers reported so far have limited selectivity towards neutrophil granules. For instance, the fusion protein containing the syntaxin-4 SNARE domain (TAT-STX-4) inhibited degranulation of all four neutrophil granule subsets in vitro, whereas the fusion protein containing the N-terminal SNAP-23 SNARE domain (TAT-SNAP-23) inhibited formyl-Met-Leu-Phe-evoked degranulation of specific granules, gelatinase granules, and secretory vesicles, but not azurophilic granules [171]. Intriguingly, TAT-SNAP-23 was found to attenuate acute lung injury induced by pulmonary immune complex deposition without altering pulmonary neutrophil accumulation in rats [155]. Subsequently, TAT-SNAP-23 administration was shown to reduce neutrophil accumulation into the lungs and lung injury in mice with sepsis- or shock-induced acute lung injury in mice [156]. Since SNAP-23 is ubiquitously expressed, further studies are needed to evaluate the effects of TAT fusion proteins on membrane trafficking in other cell types both in vitro and in vivo.

An alternative approach to block degranulation or to target MPO signaling through CD11b is the use of specialized pro-resolving lipid mediators, lipoxin A_4_ and its aspirin- or statin-triggered 15-epimeric form, 15-epi lipoxin A_4_ [174]. These lipids act through the lipoxin A/formyl peptide receptor 2 (ALX/FPR2), reduce the expression of CD11b [174,175,176] and disrupt the MPO-centered self-amplifying loop, and redirect human neutrophils to apoptosis [84]. The therapeutic potential of these lipids is illustrated by the observations of 15-lipoxin A_4_ promotion of the resolution of MPO-induced acute lung injury [84], experimental asthma [177], peritonitis [178,179], cystic fibrosis [180], ischemia-reperfusion injury [181], and bacterial pneumonia in mice [39].

## 12. Targeting NET and Silencing the MPO Autoantigens

Since aberrant NET formation contributes to the pathophysiology of many diseases, preventing NET formation or accelerating NET clearance opens potential avenues for therapy. Preclinical studies showed that MPO inhibitors could reduce NET extrusion both in vitro and in vivo, and attenuate tissue damage [144,157]. These actions were comparable to those of ROS scavengers, such as N-acetyl cysteine [157] or peptidyl arginine deiminase (PAD) inhibitors [159,160,182] in murine models of arthritis, atherosclerosis, and lupus. Studies in PAD4-knockout mice suggest that bacterial infections may shift the balance of the protective and deleterious effects of NETs in host defense [183,184]. The possible role of MPO in this shift has not been explored.

Extracellular acidification has been reported to inhibit superoxide formation [185,186] and ROS-dependent NET formation without affecting phagocytosis or bacterial killing [187]. While these actions may imply limiting tissue damage, the relevance of therapeutic induction of extracellular acidosis is uncertain.

Select specialized pro-resolving lipid mediators, such as resolvin D4, can also limit NET extrusion and consequently the formation of clots in murine models of deep vein thrombosis [188]. Recently, T-series resolvins (RvTs), which originate from n-3 docosapentaenoic acid, were identified as potent inhibitors of extracellular DNA and DNA-bound MPO in human blood [162]. These actions were detectable at nanomolar concentrations with RvT1 being the most potent one. However, additional studies are required to explore the mechanisms by which resolvin D4 and RvT1 signaled to attenuate the liberation of nuclear DNA and release of MPO (and other proteins) from the azurophilic granules. Importantly, RvTs markedly reduced *S. aureus*-triggered NET formation that coincided with decreased neutrophil accumulation and bacterial titers in mouse dorsal air pouches [162], indicating efficient control of bacterial infection. Furthermore, RvTs activated the cAMP-PKA-AMPK signaling pathway, which mediates phagocytosis and NET uptake in M0 macrophages in vitro and by peritoneal macrophages in mice [162], thereby limiting damage to neighboring tissues. 

Another potential approach is to facilitate the degradation of NETs and enhance their engulfment by macrophages [189]. Destruction of the NET scaffold with exogenous DNase 1 was reported to attenuate tissue damage and mortality in tumor-bearing mice [190], lupus-prone mice [191], acid inspiration-induced lung injury [192], and transplantation-associated lung injury in mice [193]. The synthetic DNase 1 analog dornase-α is currently being tested in a phase III clinical trial in patients with severe trauma-associated respiratory failure [194].

Persistent NET formation and NET-mediated transfer of neutrophil granule antigens (MPO and proteinase-3, in particular) to dendritic cells have been suggested to contribute to ANCA induction and associated autoimmune vasculitis [195,196,197]. Hence, targeting aberrant NET formation and/or degradation appears to be a promising approach for silencing the MPO (and proteinase-3) antigen. The pan-PAD inhibitor Cl-amidine was found to suppress the anti-thyroid drug, propylthiouracil-induced MPO-ANCA production in mice [161]. Since these mice did not develop vasculitis, the impact of PAD blockade on disease development cannot be evaluated. Studies on renal biopsy samples documented the deposition of free MPO in the glomeruli of patients with ANCA-associated crescentic glomerulonephritis [139]. MPO has been implicated in mediating NET formation and ANCA-associated endothelial damage. Consistently, the MPO inhibitor AZM198 significantly attenuated these events and the severity of crescent formation, glomerular macrophage accumulation, and antigen-specific T-cell reactivity in a murine model of nephrotoxic nephritis [139]. However, additional studies are required to assess whether MPO inhibitors could suppress the production of pathogenic auto-antibodies similar to that observed with PAD inhibitors in models of lupus [198], collagen-induced arthritis [159], and colitis [199]. 

## 13. Concluding Remarks

In addition to its well-characterized microbicidal and tissue-damaging actions, MPO is increasingly being recognized as an important mediator of neutrophil orchestration of innate and adaptive immunity. The emergence of novel paradigms in MPO functions also provides a different vantage point of the role of neutrophils in host defense, from inflicting local tissue damage and systemic complications to mediating tissue repair. Hence, MPO likely plays both pro-inflammatory and anti-inflammatory roles in a context-dependent fashion. There is a growing appreciation of neutrophil heterogeneity [20,21,200,201] and emerging data identify subsets of ‘rogue’ neutrophils that appear to be specifically associated with certain pathological conditions [202]. Whether these neutrophil subsets exhibit differences in MPO content, secretion, and/or function and whether these subsets can be targeted specifically represent fascinating novel avenues for investigations. A better understanding of the apparently paradoxical effects of MPO is essential for the development of MPO-targeting therapeutic approaches. Results from preclinical models indicate that this can be accomplished by inhibiting MPO-derived oxidants or MPO release, interference with MPO signaling, and silencing of MPO autoimmunity with small molecule inhibitors, recombinant protein inhibitors, and specialized pro-resolving lipid mediators, such as lipoxins and resolvins. Ongoing clinical trials with MPO inhibitors and modulators of its biological activities (e.g., targeting NET formation and degradation) hold promise as a disease-modifying treatment. Future studies should aim to distinguish pathological conditions where therapeutic interventions aimed at MPO could limit the deleterious actions of neutrophils in inflamed tissues or enhance neutrophil-mediated tissue repair. We believe these might eventually lead to uncovering cues that distinguish conditions where MPO is a friend or foe.

## Figures and Tables

**Table 1:** Potential therapeutic approaches to target MPO.

Molecule	Species	Disease/Model	Key Mechanisms	Effects on Disease	References
*Blocking Enzymatic Activities*					
4-Aminobenzoic acid hydrazide (4-ABAH)	Rabbit	Atherosclerosis	Irreversible inhibition of HOCl production	↓ Peroxidase activity	[132]
	Mice	Lung carcinoma		↓ Tumor progression	[133]
	Mice	Ischemic stroke		↑ Cell proliferation and neurogenesis	[69]
Polyamine-Conjugated Piperidine Nitroxides	Bovine	Aortic endothelial cells	↓ H_2_O_2_↓ HOCl, •NO_2_ scavenging	↓ Endothelial HOCl ↓ Protein nitration, ↓ NO oxidation	[134]
AZM198 (2-thioxanthin)	Mice	Obesity and hypertension	Irreversibly inhibition by covalent attachment to the heme group	↓ Body weight ↓ Fat accumulation, ↓ Inflammation ↓ Non-alcoholic steatohepatitis	[135]
	Mice	Vascular inflammation		↑eNOS/NO	[136]
	Mice	Peritonitis		↓ Tissue damage	[137]
	Guinea pig	Chronic obstructive pulmonary disease		↓ Tissue damage and remodeling	[138]
	Mice	Nephritis		↓ MPO deposition ↓ Glomerular damage	[139]
AZD3241 (Pyrrolo (3, 2-d) pyrimidin-4-one derivative)	Human	Parkinson’s disease	Selective and irreversible MPO inhibitor	↓ Neuro-inflammation	[140]
	Mice	Multiple system atrophy		↓ Microglial activation and motor impairment	[141,142]
	Mice	Colitis		↓ Weight loss ↑ Clinical score	[143]
PF-1355 (Thiouracil derivative)	Mice	Small vessel vasculitis	Irreversible MPO inhibitor	↓ HOCl ↓ Vascular edema, ↓ Neutrophil recruitment	[144]
PF-06282999 (Thiouracil derivative)	Human		Irreversible MPO inhibitor		[145]
AZD4831	Human	Heart failure with preserved ejection fraction	Selective extracellular MPO inhibitor	↓ Morbidity and mortality	[146,147]
*Suppression of MPO gene expression*					
Atorvastatin	Human	Acute coronary syndrome	?	↓ Serum MPO	[148]
	Human	Diabetes with renal failure	?	↓ Serum MPO	[149]
All-trans retinoic acid (tretinoin)	Human	ANCA vasculitis	?	↓ Disease relapse ?	[150]
*Inhibition of granule trafficking, docking and degranulation*					
Nexinhib 20	Mice	Endotoxemia	Selective inhibition of release of azurophilic granules	↓ Neutrophil infiltration	[151]
	Mice	Myocardial ischemia-reperfusion injury		↓ Neutrophil recruitment and exocytosis ↓ Infarct size	[152]
TAT-STX-4	Human	Neutrophils (*L. monocytogenes* infection)	Inhibition of degranulation	↓ Degranulation of all granule subsets	[153]
TAT-SNAP-23	Human	Neutrophils (*S. aureus* infection)	Inhibition of azurophilic granule release	↓ Exocytosis ↓ Respiratory burst	[154]
	Rats Mice	Acute lung injury		↓ Neutrophil recruitment ↓ Exocytosis	[155,156]
15-epi-lipoxin A4 and 17-epi-resolvin D1	Human		Activation of ALX/FPR2	↓ MPO release ↑ Phagocytosis and bacterial killing	[39]
	Mice	Acute lung injury		↑ Bacterial clearance ↑ Resolution	[39]
*Targeting NETs and MPO auto-antigens*					
4-Aminobenzoic acid hydrazide (4-ABAH)	Human	Neutrophils		↓ NET formation	[157]
Deoxyribonuclease I (DNase I)	Human Mice	Bacterial infection	NET degradation	↓ Reactive oxygen species	[158]
N-a-benzoyl-N5-(2-chloro-1-iminoethyl)-L-ornithine am-ide (Cl-amidine)	Mice	Rheumatoid arthritis	Peptidyl arginine deiminase (1-4) inhibitor	↓ Disease severity	[159]
	Mice	Atherosclerosis		↓ Lesion area ↓ Neutrophil and macrophages recruitment	[160]
	Mice	ANCA vasculitis		↓ MPO-ANCA production	[161]
13-series resolvins (RvTs)	Human	Neutrophils		↓ MPO release ↓ NET formation ↑ NET degradation	[162]
	Mice	Bacterial infection (Skin air pouch)		↓ Neutrophil accumulation ↑ Bacterial clearance	[162]

ALX/FPR2, lipoxin A receptor/formyl peptide receptor 2; ANCA, anti-neutrophil cytoplasmic antibody; eNOS, endothelial nitric oxide synthase; NET, neutrophil extracellular traps; Nexinhib20, neutrophil granule-specific exocytosis inhibitor 20; RvTs, 13-series of resolvins. ↓ decrease; ↑ increase; ? not defined
